# Pathological crystal structures

**DOI:** 10.1107/S2053229623007088

**Published:** 2023-10-24

**Authors:** Kenneth N. Raymond, Gregory S. Girolami

**Affiliations:** a Department of Chemistry, University of California, Berkeley, California 94720, USA; b School of Chemical Sciences, University of Illinois at Urbana Champaign, Urbana, Illinois 61801, USA; University of Bath, United Kingdom

**Keywords:** crystallographic errors, atom misassignments, incorrect modeling, disordered guest mol­ecules, incorrect space group, incorrect cell size, checkCIF

## Abstract

Recent decades have seen enormous changes in the technology of crystal structure analysis, but the inter­pretation of these data still depends on human judgment, and errors are far from uncommon. Although analysing the crystallographic results with available software tools can catch many types of errors, others can be detected only by combining knowledge of both crystallography and chemistry. We discuss several such examples from the published literature, and for each of them we identify what lessons they teach us. The examples are categorized by the type of error: correct crystallography but incorrect chemistry, mis-assignment of atoms, high-symmetry superstructures with included guest mol­ecules, incorrect choice of space group, incorrect choice of unit-cell size, and unresolved problems. These examples are intended to counteract the aura of infallibility that crystal structures sometimes assume and to alert the reader to features to look for in detecting pathological structures.

## Abstract 2

Although analysing crystallographic results with available software tools can catch many types of errors, others can be detected only by combining knowledge of both crystallography and chemistry. We discuss several such examples from the published literature, and for each of them we identify what lessons they teach us.

## Introduction

This article is intended to help students and practitioners of single-crystal X-ray structure analysis to identify and diagnose structures that may be pathological, *i.e.* incorrect or mis­leading. Both of the authors have taught hands-on X-ray structure analysis courses for many years (KR since the 1970s and GG since the 1990s), and one of us has written a textbook, *X-ray Crystallography* (Girolami, 2016 ▸). The thesis of these courses is to teach chemists (and scientists from related fields) the tools of X-ray diffraction structure analysis.

Recent decades have seen enormous increases in the quantity and speed of data collection and in the automation of structure analysis using commercial software, and so the technological parts of such courses have evolved greatly with time. However, the inter­pretation of crystallographic data still depends on human judgment: the final structural model is the product of the experimental data and the crystallographer’s expectations (and the resulting decisions they make). Sometimes the decisions the crystallographer makes are wrong. Some kinds of errors are quite common, as has been discussed over the years by others (Ibers, 1974 ▸; Donohue, 1974 ▸; Jones, 1984 ▸; Parkin, 1993 ▸; Marsh, 1995 ▸; Harlow, 1996 ▸; Fanwick, 2016 ▸; Schwalbe, 2018 ▸). Note that incorrect crystal structures often have good *R* values (least-squares residuals) and that these values are of little or no use in deciding whether a structure is correct, although they may help to decide which of two possible structural models is ‘more correct.’

Today, there are programs such as *PLATON* that serve as excellent crystallographic error-detection tools. In fact, the *checkCIF* functionality within *PLATON* forms part of the IUCr Small Mol­ecule Crystal Structure Validation facility, and many journals require that crystal structures be assessed by this tool before they can be approved for supplementary deposition (Spek, 2020 ▸). These programs can inspect the results of a crystal structure analysis and point out problems, such as unreasonable displacement parameters, unusual or inconsistent bond lengths, impossible intra- or inter­molecular non-bonded contacts, and inexplicable electron-density peaks not assignable to any atom. They can also detect when the crystal structure has higher symmetry than is embodied in the chosen space group, and suggest which higher-symmetry space group should be chosen instead.

But many kinds of crystallographic problems cannot be detected by these programs, especially those that require the author (or the reader) to think like a chemist. In many cases, the problem is that the proposed structural model is incompatible with known or expected chemical behavior. In other cases, the crystal suffers from problems that are too subtle for current automated detection tools. In the present article, we give examples of these kinds of mistakes in crystal structure analysis, and we conclude each example with advice for the reader. In some cases, the problematic structures have been amended by published corrections or retractions. Unfortunately, in many more cases, there is no published correction but the relationship to established erroneous structures and our own judgement leads us to include them as probably in error and illustrative of a flawed analysis. In many of those uncorrected cases, we made efforts in advance of writing this article to contact the corresponding authors and discuss our suspicions.

## Examples of problematic crystal structures

### Correct crystallography but incorrect chemistry

When inter­preting the results of any crystal structure, the crystallographer and the chemist must work together (if they are different people). Not all those who carry out crystal structure analyses are experts in the relevant chemistry, and most chemists are not experts in crystallography. Here we give some examples in which the crystallographer did everything correctly, but the chemist did not fully understand the implications of the crystallographic findings.

• *Sulfinato coordination of triplet-state cobalt(III) through sulfur: crystal and mol­ecular structure of bis{2-[(2-pyridyl­meth­yl)amino]­ethyl­sulfinato}cobalt(III) perchlorate dihydrate*

Many crystal structures of cobalt(III) complexes have been reported, and here we focus on one [Cambridge Structural Database (CSD; Groom *et al.*, 2016 ▸) refcode PMAESC] in which the cobalt center is coordinated to a pair of tridentate ligands, each bound *via* one secondary amine, a pyridyl amine, and the S atom of a sulfinate (SO_2_ *R*) group (Lundeen *et al.*, 1978 ▸). This cobalt(III) com­pound is a classic octa­hedral Werner complex and should be diamagnetic. However, the article states ‘The cobalt(III) sulfinato complex is paramagnetic, with Co^III^ in an inter­mediate spin state (μ = 3.27, as determined by the Evans NMR method); the occurrence of this unusual spin state is attributed to the presence of *d* 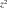 and *d*_*xy*_ levels of comparable energy as a result of electronic distortion.’ According to ligand field theory, a change in spin state would be expected to lengthen some of the cobalt–ligand bond distances, but the bond lengths conform perfectly to those seen in many hundreds of low-spin cobalt(III) com­plexes.

What is the answer to this conundrum? The Evans NMR technique involves measuring the change in an NMR chemical shift (usually of a solvent peak) caused by the paramagnetism of a dissolved com­pound. The cobalt(III) com­pound had been prepared from a cobalt(II) starting material, which could have been the source of the paramagnetism in the Evans mea­sure­ment. Alternatively (and we think, more likely), perhaps an impurity peak was mistaken for a paramagnetically-shifted peak due to the reference. Whatever the reason for the dis­cre­pancy, it was not realized that the crystallographic results and the Evans measurement were contradictory.

**Lesson:** authors should critically assess whether the crystallographic results are consistent with other information about the compound of interest. Sometimes the discrepancy arises because the other information is incorrect.

• *Synthesis of optically active 2*H*-thio­pyrano[2,3-*b*]quinolones with three contiguous stereocenters via an organocatalytic asymmetric tandem Michael–Henry reaction*

The quinoline scaffold is prevalent in a variety of pharmacologically active synthetic and natural com­pounds, and so there is great inter­est in the asymmetric synthesis of such chiral com­pounds. In one article reporting such a com­pound (SERNAG), the authors state that ‘The relative and absolute configuration of the product **5ad** is unequivocally established by X-ray analysis (Fig. 3), and the remaining configurations are assumed by analogy’ (Wu *et al.*, 2013 ▸). Fig. 3 in the article shows a picture of the chiral mol­ecule, with its three stereocenters, established as 2*R*,3*R*,4*S*.

The reported space group of the quinolone crystal was *P* 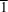 (*i.e.* primitive triclinic with an inversion center), with two mol­ecules per unit cell. There is a problem with this! An inversion center inter­relates mol­ecules of opposite chirality; it is impossible that only one chiral isomer was present.^1^ The authors corrected their error (POJLUX) with the statement: ‘Recently, Nicholas Hext, a Scientific Information Analyst at Chemical Abstracts Service in Columbus, OH, USA, informed the authors that the analysis of CCDC 927204 gives data for the racemic com­pound (Wu *et al.*, 2014 ▸).’

**Lesson:** all centrosymmetric space groups require that the crystal consist of an equal mixture of both the left- and right-handed enantiomers. Good communication between the chemist and the crystallographer, or a good understanding of crystallography by the chemist, is crucial to reaching correct conclusions about the compound under study.

### Wrong identification of atoms

Atoms with similar atomic numbers scatter X-rays very similarly, and sometimes this phenomenon leads the crystallographer to identify an atom incorrectly. A very rough rule of thumb is that atoms whose atomic numbers differ by less than 10% cannot easily be distinguished. Thus, the atomic numbers of nitro­gen (*Z* = 7) and oxygen (*Z* = 8) differ by 15% and may be distinguishable, whereas the 7% difference between tungsten (*Z* = 74) and gold (*Z* = 79) will make it difficult to tell them apart. For example, several crystal structures (including one published in *Science*) of polyoxometalate anions thought to contain gold–oxo, palladium–oxo, or platinum–oxo bonds were retracted because in each case the supposed late transition metal was actually tungsten (Anderson *et al.*, 2012*a* ▸,*b* ▸; Cao *et al.*, 2012 ▸; O’Halloran *et al.*, 2012 ▸). And, for a natural product having anti­neoplastic activity, the mis-assignment of an NH group as oxygen in a crystal structure (JIMBUC), along with some NMR mis-assignments, caused several research groups to spend years of effort trying to make the wrong com­pound (Li *et al.*, 2001 ▸; Wilson, 2001 ▸).

One common symptom of an incorrect atom type is that the bond distances to that atom are incorrect; another is that the displacement parameters for that atom are inexplicably different from those of nearby atoms (Amemiya *et al.*, 2020 ▸; Shlian *et al.*, 2023 ▸). But other factors can also affect displacement parameters, such as thermal motion, disorder, and an incorrect site-occupancy factor, so that the displacement pa­ram­eters cannot always indicate the presence of mis-assigned atoms. Sometimes, wrongly identified atoms can be discovered only because the identity of an atom does not make chemical sense, or contradicts other experimental data, as in the polyoxometalate species above. Here we focus on several other examples that illustrate this point.

• *Crystallographic snapshot of an arrested inter­mediate in the biomimetic activation of CO_2_*

**Figure 1:**
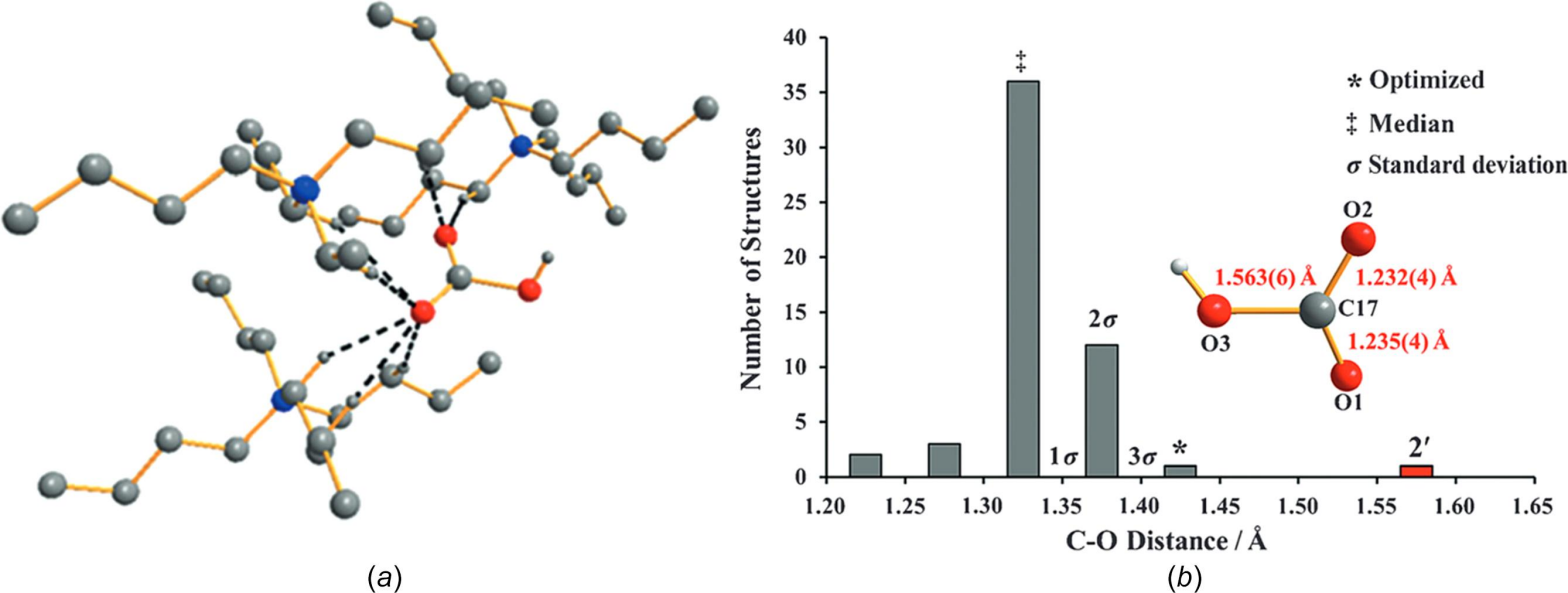
(*a*) Ball-and-stick diagram showing the supposed CO_2_ mol­ecule being attacked ‘incipiently’ by a hydroxide ion (O atoms depicted as orange spheres). (*b*) Histogram taken from literature crystal structures showing that the C17—O3 distance is much longer than normal C—O distances, but without realizing that O3 is actually the C atom of a methyl group. (Reproduced with permission of Wiley.)

The activation of carbon dioxide is a topic of considerable inter­est owing to the issues of global warming and the carbon cycle. In one article, the reaction of tetra-*n*-butyl­ammonium hydroxide with CO_2_ was claimed to result in ‘an unprecedented arrested inter­mediate’ and ‘a significant discovery’ (Ackermann *et al.*, 2015*a* ▸). Specifically, they stated that: ‘We conclude that the structure of the anion in **2′** represents a unique experimental snapshot of the incipient binding of CO_2_ by the hydroxide ion, which is both assisted and restrained by unconventional hydrogen-bonding inter­actions with the hydro­phobic cavity formed by the tetra­alkyl­ammonium cation scaffold.’ In their structural model (FOVBOJ; Fig. 1 ▸), a CO_2_ mol­ecule is bent from its usual linear geometry [the O—C—O angle is 126.9 (3)°] and, even more surprisingly, the C atom forms an unusually long C⋯O distance of 1.563 Å to an attacking hydroxide group. Normal C—O bond distances are much shorter than this, almost always less than 1.40 Å.

This report was followed by an article that disputed its findings: ‘[We] believe that … the authors have synthesized and spectroscopically characterized powdered bicarbonate, [*n*Bu_4_N][HCO_3_], and carried out an X-ray crystallographic study on a single crystal of acetate, [*n*Bu_4_N][O_3_CCH_3_]’ (Hur­malainen *et al.*, 2015 ▸). In other words, the unusually long C—O bond is actually a C—C bond; the 1.563 Å distance is much more consistent with this atom assignment than the original one (FOVBOJ02). This reformulation was subsequently agreed to by the original authors, who wrote a corrigendum (Ackermann *et al.*, 2015*b* ▸). Of course, a methyl group has three H atoms, *versus* one for hydroxide, and left unsaid in the correction was whether there were two suspicious unassigned electron-density peaks near the hydroxide ‘O’ atom (as there should have been).

**Lesson:** any crystal structure that has an unprecedented feature should be a warning sign that something may be wrong. In the present case, the 1.5 Å distance is unprecedented for a C—O bond but is quite consistent with a C—C bond. It is important always to consider alternative and more chemically plausible explanations of any surprising result.

• *A novel heterocyclic com­pound: *catena*-poly[[[di­aqua­sodium(I)]-di-μ-aqua] hemi(1,5-dihy­droxy-4,8,9-trioxa-2,6-di­aza­bicyclo­[3.3.1]nona-2,6-diene-3,7-diolate)]*

In 2007, *Acta Crystallographica Section C* published an article reporting the crystal structure of (according to the title) a ‘novel heterocyclic com­pound’ with the chemical formula [Na(H_2_O)_4_]_2_[C_4_N_2_O_5_(OH)_2_] (Fang *et al.*, 2007 ▸). The crystal had been obtained from the reaction of sodium carbonate (Na_2_CO_3_) and ammonia (NH_3_) in water at 140 °C in a sealed vessel.

**Figure 2:**
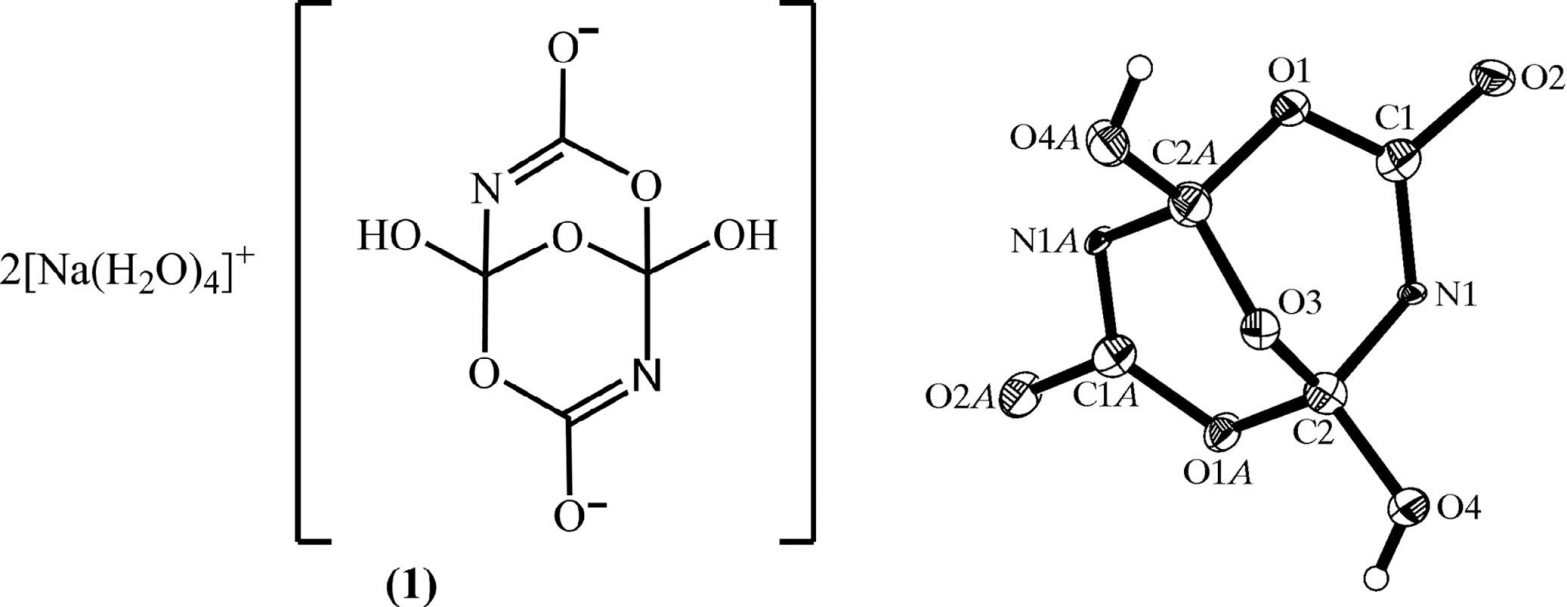
Example of multiple atom mis-assignments. (Reproduced with permission of the Inter­national Union of Crystallography.)

The crystal structure (Fig. 2 ▸) revealed the presence of a grouping of C, N, and O atoms arranged into two fused six-membered rings. The authors did not comment on the fact that the displacement parameters of the N1 atom (and its symmetry-related counterpart N1*A*) were unusually small compared to those of the atoms in its vicinity. There can be many reasons for this type of behavior, but one is that the identity of the atom has been chosen incorrectly, and that the atom actually has a higher atomic number. Both N atoms are in fact O atoms, but this is not the only atom mis-assignment. All of the atoms identified as carbon are actually boron, and the sample studied was not a novel organic heterocycle, but instead a crystal of the well-known mineral borax, [Na(H_2_O)_4_]_2_[B_4_O_5_(OH)_4_] (Fang *et al.*, 2008 ▸). The article was subsequently retracted by the authors (Fang *et al.*, 2008 ▸).

Inter­estingly, none of the reactants used in the preparation of the sample contained boron. The article does not specify the composition of the reaction vessel, but one possibility is that it was made of borosilicate glass, out of which the boron was leached at the high temperatures under which the crystal was grown. The mis-assignment of boron as carbon (and oxygen as nitro­gen) in both cases involves elements that differ by only one in atomic number.^2^

**Lesson:** unusually small (or large) displacement parameters are alerts that something may be wrong with the atom assignments. Alternative assignments should be considered to test whether they lead to more consistent displacement parameters.

• A novel, linear, two-coordinate Rh^I^ anionic amide complex formed by the reaction of the nucleobase 1-methyl­thymine with the [(Cp*Rh)_2_(μ-OH)_3_]^+^ cation at pH 10: mol­ecular recognition and electrostatic inter­action within an organometallic hydro­phobic cavity

**Figure 3:**
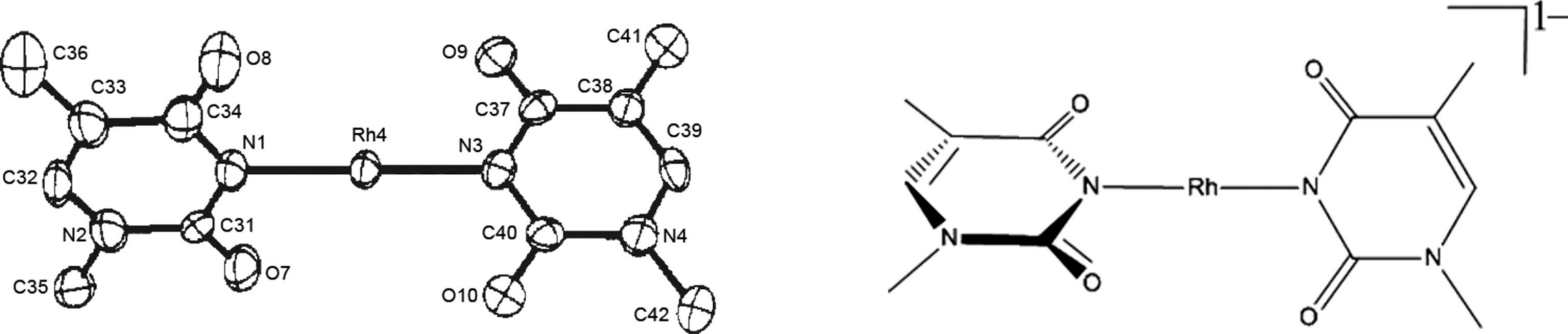
The reported two-coordinate rhodium(I) com­pound that we are convinced is actually a silver(I) com­pound. (Reprinted with permission of the American Chemical Society.)

An effort to make organometallic complexes of DNA bases led to the report of the crystal structure of a com­pound (ZEKYET, later republished as ZOLFOX) claimed to be ‘a novel, linear, two-coordinate rhodium(I) anionic amide’ (Chen *et al.*, 1995 ▸; Smith *et al.*, 2014 ▸). The com­pound had been obtained by the reaction of the rhodium penta­methycyclo­penta­dienyl complex [C_5_(CH_3_)_5_]RhCl_2_ first with a silver salt (to remove the chloride ligands) and then with 1-methyl­thymine in a basic solution. According to the article, the crystal structure showed that the product contained a rhodium(I) ion bearing two deprotonated 1-methyl­thymine ligands in a linear two-coordinate arrangement; the C_5_(CH_3_)_5_ groups had been lost (Fig. 3 ▸). The Rh—N bond length was 2.09 Å.

The microanalysis for carbon, hydrogen, and nitro­gen fit the chemical formula suggested by the crystallographic results, and the spectroscopic data suggested that no metal hydride ligands were present. But several things about the chemistry were unusual. First, penta­methyl­cyclo­penta­dienyl rings, C_5_(CH_3_)_5_, bind very strongly to metal centers and are seldom lost in mild reactions; second, no other two-coordinate com­pounds of rhodium have ever been described (before or since).

Although this crystal structure has not been corrected, we are convinced that the ‘Rh’ atom is actually silver. Many linear two-coordinate silver(I) com­pounds are known, and rhodium and silver are only two places apart from one another in the Periodic Table (the atomic numbers differ by 4%). Here, silver had been added in a previous step to precipitate chloride, but the assumption was made (incorrectly) that no silver could have been incorporated into the data crystal.

**Lesson:** whenever a crystal structure suggests a structure that has no literature precedent, it is well worth considering other possible explanations.

• *Ni_2_[LnCl_6_] (Ln = Eu^II^, Ce^II^, Gd^II^): the first Ln^II^ com­pounds stabilized in a pure inorganic lattice*

In 2018, three crystal structures were reported (ICSD collection codes 1813231, 1813639, and 1813640) of com­pounds claimed to have the formula Ni_2_[LnCl_6_], where the lanthanide metal was cerium, europium, or gadolinium (Baldo *et al.*, 2018 ▸). These com­pounds were prepared solvothermally by the reaction of nickel dichloride and the appropriate lanthanide trinitrate with a carb­oxy­lic acid and tri­methyl­amine in di­methyl­formamide solvent at 170 °C. The article claims that the lanthanide ions had been reduced to the divalent state, but this redox change is always extremely difficult (MacDonald *et al.*, 2013 ▸) and certainly impossible under the given reaction conditions. Furthermore, the crystals survived for long periods in air, whereas divalent lanthanide complexes rarely if ever exhibit this property.

In addition, the 2.1 Å metal–ligand distances in the com­plexes studied are far too short for any Ln—Cl bond: the Ln—Cl distances seen for Ln^III^ ions are typically about 2.7 Å, and those for divalent lanthanide ions should be even longer. Furthermore, the Ni atoms are not coordinated to any other atom, and instead seem to serve as counterions. No com­pounds are known in which nickel behaves like this. So what are these com­pounds really?

The article was subsequently retracted, but the retraction is brief and relatively uninformative (Baldo *et al.*, 2019 ▸). Here we fill in the details. Notably, all three com­pounds are isomorphous with crystals of the nickel ammonia complex [Ni(NH_3_)_6_]Cl_2_ (Essmann *et al.*, 1996 ▸; Wagner *et al.*, 2000 ▸), although all three lanthanide complexes had unit cells that were larger than for this nickel com­pound by 0.1 Å along each axis (too large a difference to be attributable to random error). The purple color of all three com­pounds, however, exactly matches the color of the [Ni(NH_3_)_6_]^2+^ ion. The Ni—N distance in the [Ni(NH_3_)_6_]^2+^ ion of 2.12 Å is almost identical to the 2.13–2.16 Å distances reported as supposed lanthanide–chloride bonds. Thus, the ‘LnCl_6_’ units are actually [Ni(NH_3_)_6_]^2+^ ions.

This reformulation also accounts for the magnetism and the electronic spectra. The discrepancy in the unit-cell parameters *versus* that for [Ni(NH_3_)_6_]Cl_2_ can be explained if the counteranion is not chloride alone, but a mixture of chloride and nitrate. From a crystallographic viewpoint, the reformulation also makes sense, because all the atoms in this structure have been assigned atomic numbers that are too large by a little over a factor of two: *Z*(Ln) ≃ 65 *versus* *Z*(Ni) = 28 (ratio = 2.32); *Z*(Cl) = 17 *versus* *Z*(N) = 7 (ratio = 2.42); *Z*(Ni) = 28 *versus* an equal mixture of *Z*(Cl) = 17 and *Z*(N) = 7 (ratio = 2.33). This is what would happen if the overall scale factor relating *F* ^2^(obs) and *F* ^2^(calc) had been set to a value that is wrong by about a factor of 5.5 (= 2.35^2^): the incorrect scale factor makes all the electron-density peaks appear larger than they actually should be. What remains unclear is where the ammonia in the crystals came from, because the reported procedure contains no obvious source of this ligand. Finally, we note that, had a microanalysis been carried out, it would have immediately revealed that the proposed molecular formulae were incorrect.

**Lesson:** crystallographic results should make chemical sense: the bond distances should be consistent with those in related compounds, the chemical and physical properties should be consistent with those expected for the chemical model that was used to fit the crystallographic data. If in doubt, collect additional data to prove whether the proposed model is in fact correct.

• *(Octa­ethyl­aza­porphyrinato)iron(III) chloride: its structure in the solid state and in solution*

Often, when carrying out a crystal structure analysis of a mol­ecule of inter­est, it is found that the mol­ecule has formed a cocrystal with a second substance (such as a solvent), in which case the chemist/crystallographer must decide what second species is present. A crystal structure (JUXNOF) was once reported claiming that an iron porphyrin com­pound had cocrystallized with di­nitro­gen mol­ecules, N_2_; this is unusual enough (small mol­ecules easily escape from crystals and are rarely trapped unless they are strongly bound), but even more surprisingly, the latter had dimerized into N_4_ units (Balch *et al.*, 1993 ▸). This dimerization was not noticed in the original article, however, because the analysis of the model had been restricted to just a single asymmetric unit, and evidently there had been no search for additional interatomic contacts. An article reporting a reinvestigation (JUXNOF10) noticed the ‘dimerized N_2_ mol­ecule’ and stated that if the finding were true ‘it would be a remarkable discovery, worth celebrating as a milestone in synthetic and structural chemistry and in nitro­gen fixation’ (Marsh *et al.*, 1993 ▸). The reinvestigation clearly showed, however, that the electron density could be inter­preted as a disordered mol­ecule of di­chloro­methane, the solvent from which the iron porphyrin com­pound had been crystallized. The electron density for each Cl atom (*Z* = 17), disordered over two sites, would be similar to that of one N atom (*Z* = 7).

**Lesson:** when analysing a crystallographic model, it is im­por­tant to consider inter­atomic inter­actions that extend outside of the asymmetric unit, and an analysis of the entire unit cell should be carried out. Such an assessment can sometimes lead to the realization that the proposed model is chemically impossible.

### High-symmetry superstructures with included guest mol­ecules

A fundamental property of crystallography is that the electron-density distribution around a so-called special position must have the symmetry imposed on that position by the space group. Thus, the electron-density distribution within the pores of a high-symmetry superstructure will have the symmetry of that position. Almost always, this means that the contents of the pores are disordered, so that the inter­pretation of the apparent electron density will lie in the eye of the beholder. Often, the electron density is overinter­preted, when in reality very little structural information can be deduced with certainty. The resulting structural model may well have exciting aspects worthy of publishing in the most prestigious journals, but in fact the model is fantasy rather than reality. Sadly, this error occurs persistently and frequently.

• *Zero-coordinate Rb^+^: a rubidium ion whose inter­ionic contacts are all unconventionally long by more than 1.5 Å*

Zeolites are nanoporous aluminosilicates that find wide use, for example, as drying agents and as key components in several important catalytic processes. One attractive feature of zeolites is that the water and sodium counterions, which occupy the pores of many as-prepared zeolites, can be replaced by many other chemical species. Although the aluminosilicate framework is usually well-ordered crystallographically, the contents of the pores usually are not. Inter­pretation of the electron density inside the pores requires some care and judgment.

From one crystal structure of a zeolite 4A that had been exchanged with rubidium cations (ICSD collection code 200026), the authors concluded that there was evidence for ‘the existence of an uncoordinated Rb^+^ ion in Rb^+^-exchanged zeolite’ (Firor & Seff, 1976 ▸, 1977*a* ▸). Now, except for certain noble gas clathrates, zero-coordinate atoms and ions are never seen in the solid state. The finding (if true) would be astonishing, par­ticularly in view of the expectation that the positively charged rubidium ions should be strongly attracted to the negatively charged O atoms of the zeolite pores in which they reside.

This article (and several others by the same group) was later rebutted with the statement that ‘All the X-ray structure refinements made by Seff and colleagues have technical problems: (*a*) there is no independent determination of the chemical composition of the crystal used for X-ray analysis, (*b*) the supposed zero-coordinated cations are represented by irregular or weak electron-density peaks similar in size to residual peaks not ascribed to atoms, and (*c*) refinement was made for the pseudostructure (*a* = 12 Å, *Pm*3*m*) rather than the superstructure with ordered Al and Si atoms… The simple but important conclusion is the lack of valid evidence for zero-coordinated [ions] in dehydrated… zeolite. The second conclusion is that all the cations lie adjacent to framework oxygen atoms’ (Pluth & Smith, 1979 ▸). Sadly, the story continued in several iterations. For example, an article (Subramanian & Seff, 1977 ▸) claiming the presence of zero-coordinate sodium inside zeolite A (ICSD collection code 200253) was followed by a decisive rebuttal (Pluth & Smith, 1980 ▸). Many more examples of over-inter­preted electron density inside of zeolite A crystals have never been explicitly rebutted, but which are nonetheless almost certainly incorrect.^3^

**Lesson:** The symmetry of disordered material inside a superlattice structure will have the symmetry assigned by the space group of that lattice. Care should be taken not to let one’s imagination exceed what can actually be seen.

• *Single-crystal X-ray structure of 1,3-di­methyl­cyclo­butadiene by confinement in a crystalline matrix*

**Figure 4:**
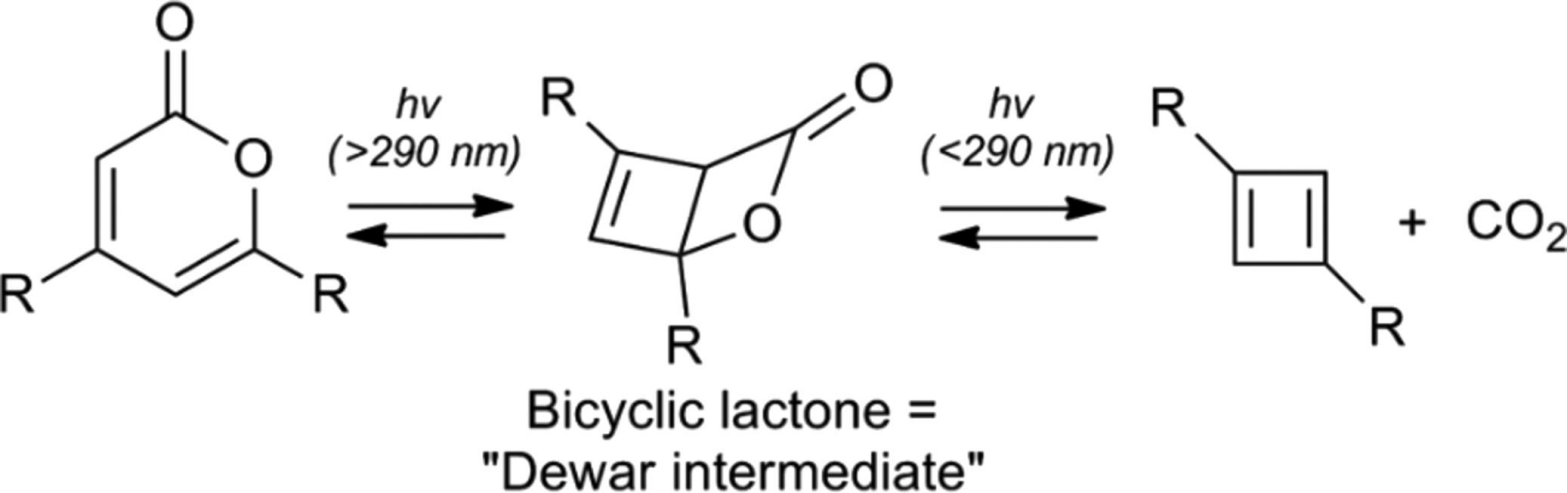
Supposed photochemical preparation of a cyclo­butadiene from α-pyrone. (Reproduced with permission of Wiley and the Deutsche Chemische Gesellschaft.)

Cyclo­butadiene (CBD) is the classic anti­aromatic com­pound, having 4*n* π electrons as opposed to the 4*n*+2 π electrons in aromatic com­pounds such as benzene. As a result, cyclo­butadiene is not a stable mol­ecule, and no crystal structure of it is known [although there are some structures of tetra­sub­sti­tuted cyclo­butadienes (Irngartinger & Nixdorf, 1988 ▸; Irn­gartinger *et al.*, 1988 ▸)]. In 2010, a vigorous scientific debate began when a crystal containing 4,6-dimethyl-α-py­rone, im­mobilized in a guanidinium–sulfonate–calixarene (G_4_C) crystalline network, was photolyzed at low temperature (Legrand *et al.*, 2010*a* ▸). The resulting material was examined crystallographically at different photolysis times (MUWMEX and MUWMEX01), and the crystal structures showed that pyrone had been converted into a disordered mixture of com­pounds. The authors claimed that, at long irradiation times, photolysis had cleaved the pyrone into two isomers of di­methyl­cyclo­butadiene (Me_2_CDB) and carbon dioxide (Fig. 4 ▸). The article states: ‘Our data support experimental observation of square-planar (Me_2_CBD^S^) and rectangular bent (Me_2_CBD^R^) geometries in the G_4_C host matrix. The hydrogen-bonded, dissociated carbon dioxide coproduct inter­acts more strongly with Me_2_CBD^S^ than with Me_2_CBD^R^.’

This claim was subsequently rebutted by two articles (Scheschkewitz, 2010 ▸; Alabugin *et al.*, 2010 ▸), one of which stated: ‘The relatively small carbon–oxygen distances between the CO_2_ moiety and the CBD unit from 1.502 to 1.612 Å were attributed to van der Waals inter­actions even though they are fully in line with typical covalent single bonds… [and] all observed structures correspond to only one distinct species, the Dewar β-lactone.’ In other words, photolysis of the pyrone had not afforded a cyclo­butadiene, but instead the reaction stopped at the middle species in Fig. 4 ▸. The original authors subsequently reinvestigated the structure (MUWMEX02 through MUWMEX05) and defended their original conclusions (Legrand *et al.*, 2010*b* ▸, 2011 ▸, 2013 ▸), but the only new argument in this rebuttal to the rebuttal was that their inter­pretation of the disordered electron density in their crystal was no worse than those in other earlier studies of reactive host–guest crystals.

In a later article, one of the critics independently re-examined the original data set and came to a conclusion that we regard as the current definitive result (Shatruk & Alabugin, 2013 ▸). ‘While we do not repudiate the possibility of partial CBD formation in the reported experiments, we clearly show that it is impossible to claim that the structure of the CBD has been unambiguously and accurately established based on the available crystallographic data.’

**Lesson:** The modeling of disordered electron density often requires that the crystallographer/chemist impose their own inter­pretation of the identities of the disordered components. It is a significant mistake to conclude that a structural model that provides a reasonable fit to the observed disordered electron density is the only possible model; at the very least, one should explicitly evaluate and discuss whether other models may equally well fit the data, even though they might be less chemically novel or exciting.

• *A stable zirconium-based metal–organic framework for specific recognition of representative polychlorinated dibenzo-*p*-dioxin mol­ecules*

It is well appreciated that the contents of the pores of metal–organic frameworks (MOFs) are usually disordered, for many of the same reasons that pertain to zeolites and other host–guest crystals (Lee *et al.*, 2018 ▸; Allendorf *et al.*, 2021 ▸). In a *Nature Communications* article published in 2019 (Wang *et al.*, 2019 ▸), the crystal structure of a zirconium-based metal–organic framework (called BUT-17) was published (JOMZOD), in which it was proposed that the one-dimensional hexa­gonal channels and aromatic-rich pore surfaces enabled the MOF to bind specifically to 2,3-di­chloro­dibenzo-*p*-dioxin (BCDD), a representative of a large family of long-lived environmental pollutants (Wang *et al.*, 2019 ▸).

In a rebuttal article, a different group reinvestigated the crystal structure and summarized their findings as follows (Poręba *et al.*, 2022 ▸). ‘After a first inspection of the structure, one is immediately impressed by the unusually large displacement parameters (*U*_iso_) of the BCDD atoms, despite a partial occupation of only 16%, fixed without any justification. Moreover, the model does not consider the inherent disorder due to the mol­ecule sitting about an *mm*2 symmetry site. Thus, the modeled mol­ecule is actually tetrachloro­dibenzo-*p*-dioxin instead of BCDD. Based on the reported XRD data, we refined the structural model fixing the *U*_iso_ to those refined for the MOF organic linkers… The resulting occupancies drop to 1% or smaller except for C17 and C18, which have site occupation factors of *ca* 8 and 3%, respectively. This clearly shows that the electron-density peaks do not form a connected set of the modeled mol­ecule… We then removed the BCDD from the reported BCDD@BUT-17 and refined a guest-free model. The agreement indices are nearly identical to those for the model including the guest mol­ecule.’

This rebuttal, which included several evaluations of the proposed structural model that should have been carried out (but weren’t), clearly showed that the BCDD mol­ecules in the original report were mis­re­pre­sen­tations. In response to this thorough reinvestigation, the authors of the original article replied (Wang *et al.*, 2022 ▸): ‘From the crystallography point of view, we admit that the diffraction data provided in our article was not good enough to clearly show that BCDD was indeed absorbed into the pores of BUT-17.’ In the same reply, however, they reported the results of a crystal structure analysis on a new data crystal, which they say support their original claims but which in our eyes is also far from convincing.

**Lesson:** once again, be careful that your wishes are not guiding the inter­pretation of the data, especially in disordered structures.

### Incorrect choice of space group

In most published crystallographic studies in which an incorrect space group has inadvertently been chosen, the correct space group is usually a supergroup of the chosen one, which means that the true space group has additional symmetry elements. Automated tools such as *PLATON* and *checkCIF* can easily analyse a proposed crystal structure and detect the presence of supergroups. A less common situation is when the correct space group is a subgroup of the incorrect one; *i.e.* the true space group has fewer symmetry elements (Burrell *et al.*, 1995 ▸; Calabrese & Gardner, 1985 ▸; Cotton *et al.*, 1994 ▸; Kuchta *et al.*, 1996 ▸). Here we give an example of an even rarer phenomenon, in which the choice of space group falls in neither of these classes. Although the crystallographic results looked mathematically acceptable, the chemical consequences were simply unrealistic.

• *Structure of the inter­mediate iron(0) complex isolated from the di­nitro­gen fixing system LiPh + FeCl_3_*

**Figure 5:**
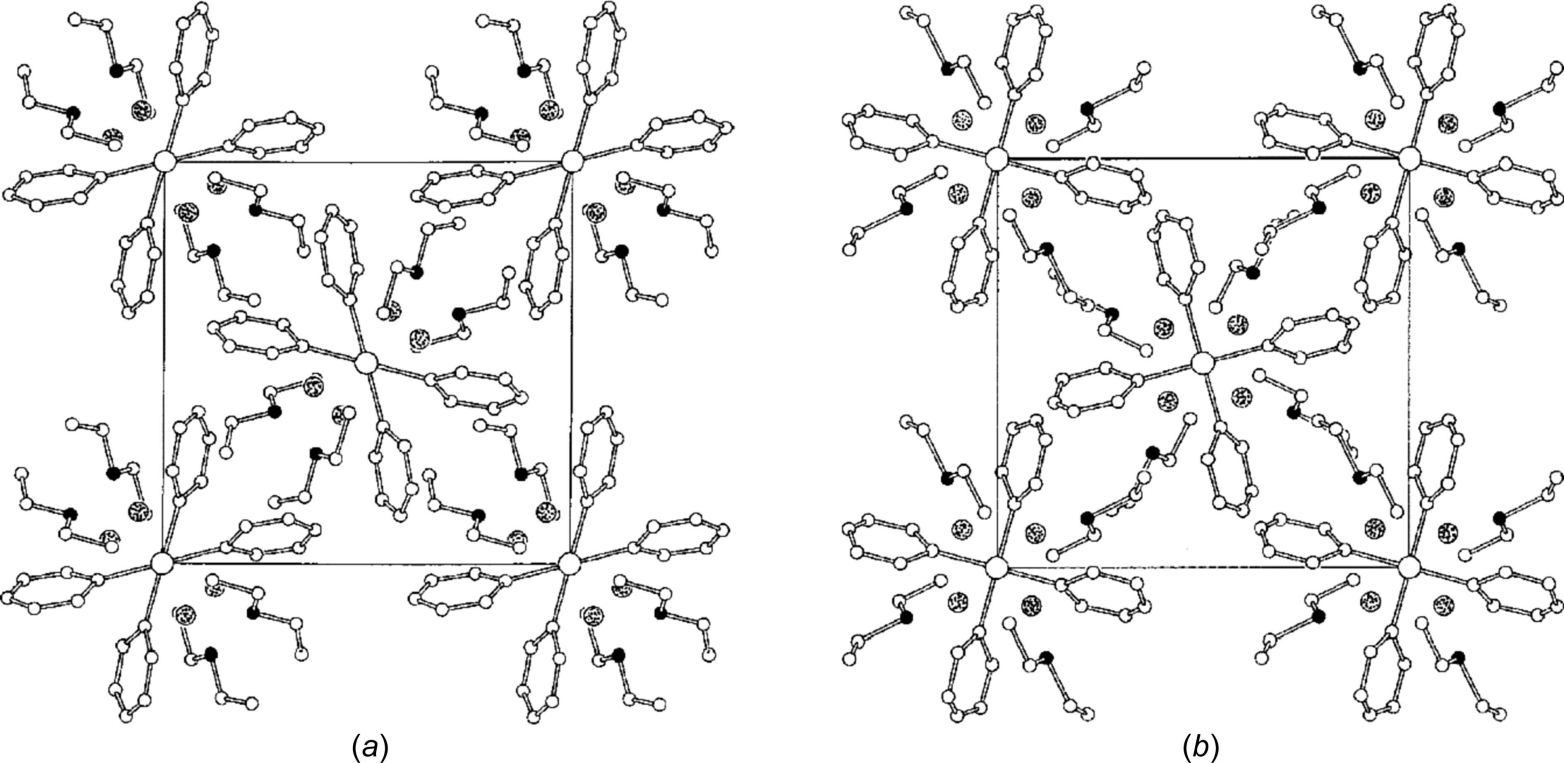
The iron structure in (*a*) *P*4_2_2_1_2 and (*b*) *P* 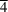 2_1_ *c*. Compare the Fe—Fe vectors, the Fe—C vectors, and the Fe—Li vectors, and recall that the Fourier transform of the diffraction intensities (*F* ^2^) is the Patterson function, in which peaks are atom-to-atom vectors as opposed to atom positions in the electron-density map. (Reprinted with permission of the American Chemical Society.)

In 1983, the crystal structure of an organoiron com­pound of stoichiometry [Li(Et_2_O)]_4_[FePh_4_] (**1**) was reported (Bazhenova *et al.*, 1983 ▸), in which the iron centre in **1** adopted an unprecedented coordination geometry described as ‘flat rectangular’: the four *ipso*-C atoms formed a planar array in which the *cis*-C—Fe—C angles were either 61 or 119° (BUJWOS; Fig. 5 ▸). A remarkably short ‘non-bonded’ C⋯C contact of 2.09 Å was said to exist between the *ipso*-C atoms of the closely situated pairs of adjacent phenyl groups.

Although the unusual structure of the complex was pro­posed to be related to its ability to activate N_2_, several aspects of the structure were not believable. First, as formulated, the iron centre is formally zerovalent and such a *d* ^8^ metal centre should adopt a regular square-planar structure with 90° inter­ligand angles. Second, the 60° C—*M*—C angles are some 20° smaller than the smallest such angle seen between two phenyl groups in any other transition-metal complex.

The erroneous structural features were artefacts of a false solution in an incorrect space group. The structure should have been solved in the space group *P* 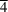 2_1_ *c* instead of *P*4_2_2_1_2 (Jefferis & Girolami, 1998 ▸, 1999 ▸). In the correct space group (BUJWOS02), the phenyl groups actually describe a regular square-planar geometry with *cis*-C—Fe—C angles that are exactly 90° (Fig. 5 ▸). Other aspects of the structure (involving the lithium ions) also make much more sense in the correct space group. Finally, the formulation of the com­pound as a derivative of zerovalent iron is incorrect: the com­pound is actually an iron(II) dihydride of stoichiometry [Li(Et_2_O)]_4_[*trans*-FeH_2_Ph_4_], in which the H atoms occupy the ‘vacant’ axial sites above and below the square plane of phenyl groups bound to the iron centre.

The mistake arose from an incorrect identification of the systematic absences: *P* 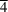 2_1_ *c* and *P*4_2_2_1_2 have many absences in common, but *P* 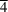 2_1_ *c* has additional absences of the form *hhl* with *h* > 0 and *l* ≠ 2*n* that *P*4_2_2_1_2 does not, or, in other words, during the original data collection some absences were missed. Unlike almost all other examples in which the wrong space group is chosen, here the two space groups *P*4_2_2_1_2 and *P* 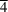 2_1_ *c* do not have a subgroup/supergroup relationship: they have (almost) completely different sets of symmetry operations. Inter­estingly, the false solution and the correct one have the same asymmetric unit, and the two crystal structures differ only in the choice of point group used to generate the three-dimensional structure from the basic structural motif.

**Lesson:** whenever a crystal structure gives a result that is structurally unprecedented, the result is probably wrong, and all other possible explanations of the unexpected result should be explored. In the present case, the resolution of the pro­blems is not an obvious one, but at the very least the original structural model should not have been published.

### Incorrect choice of unit-cell size

One of the first steps in carrying out a crystal structure determination is to choose a unit cell that accounts for the directions at which the X-ray reflections exit from a crystal. Today, determining the unit cell is almost entirely automated and requires little participation by the crystallographer. But the automated indexing and cell-refinement software routines can sometimes be fooled. For example, if the true cell consists of two halves in each of which the atoms are almost but not exactly related to one another by a pure translation of half the length of a cell axis, then certain sets of reflections become almost vanishingly weak. If the software and the crystallographer overlook these weak reflections, the chosen unit cell will be too small [by a factor of two in the example given here, but larger factors are possible (Harrowfield *et al.*, 2002 ▸)]. Refining the data in this smaller cell leads to an electron-density distribution that is an average of the two halves. This averaging will be seen as a 50:50 disorder of the atoms, which will complicate the refinement and the structural inter­pretation.

• *Discrete dinuclear cyano-bridged complexes*

**Figure 6:**
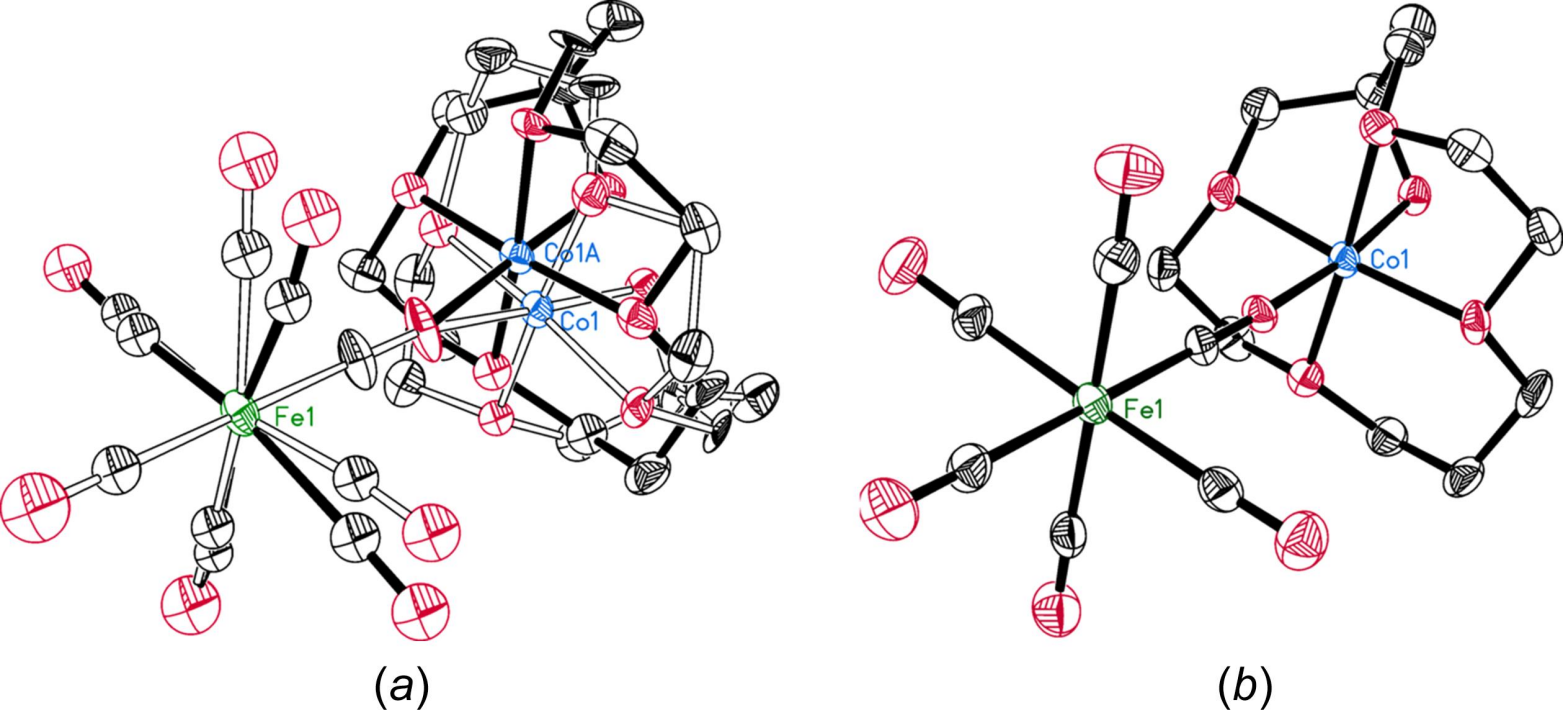
(*a*) The original structural model of *L* ^14^CoNCFe(CN)_5_ in the 10.0 × 13.3 × 10.1 Å unit cell, showing 50:50 disorder of almost all the atoms. (*b*) The final structural model in the 10.0 × 13.3 × 20.2 Å unit cell in which the mol­ecule is fully ordered. (Adapted with permission of The Royal Society of Chemistry.)

Crystals of the cyano-bridged cobalt–iron com­pound *L* ^14^CoNCFe(CN)_5_, where *L* ^14^ is a macrocycle containing five N atoms (QALQID), were found to be monoclinic with two mol­ecules in a 10.0 × 13.3 × 10.1 Å unit cell (Bernhardt *et al.*, 2000 ▸). Refinement in the space group *P*2_1_/*m* showed that 26 of the 30 non-H atoms were disordered over two sites related by a mirror plane, so that the occupancy factors of these atoms were all exactly 0.5 (Fig. 6 ▸). The *wR*_2_ factor of 0.17 for 2458 reflections and 216 parameters was relatively high, and many of the C atoms could only be refined isotropically.

Iron (*Z* = 26) and cobalt (*Z* = 27) are heavy atoms and will tend to dominate the intensities of the reflections. The Fe atoms did not appear to be appreciably disordered, and the Co atoms were disordered over two positions only 0.7 Å apart. If the unit cell had a larger supercell, only the weakly scattering C and N atoms would contribute significantly to the extra reflections, and so it would not be surprising if these extra reflections had been overlooked. Re-inspection of the dif­fraction pattern revealed that extra reflections were in fact present, and that the unit-cell dimensions were actually 10.0 × 13.3 × 20.2 Å; in other words, the *c* axis was double the original length (Bernhardt *et al.*, 2002 ▸). The space group in this larger cell was *P*2_1_/*c* (instead of *P*2_1_/*m*) and there were four mol­ecules in the unit cell (instead of two). But the mol­ecules were now fully ordered rather than disordered (QALQID01; Fig. 6 ▸). The *wR*_2_ factor was 0.12 for 4706 reflections and 316 parameters.

**Lesson:** Especially for crystals that contain a small number of heavy atoms, whenever a large fraction of the light atoms in a crystal structure are disordered with site-occupancy factors that are exactly 0.5, the presence of a supercell should be considered. In such cases, the original diffraction images should be re-examined to look for additional weak reflections that would indicate the presence of a larger unit cell. For the present example, the correct mol­ecular structure was deduced despite the choice of the wrong cell, but the bond distances and angles were highly inaccurate.

### Unresolved problems

Lastly, we consider a case in which the crystallographic results (and the chemical conclusions drawn from those results) are certainly wrong, but the reasons are still unclear.

• *A *cis*-dioxido uran­yl: fluxional carboxyl­ate activation from a reversible coordination polymer*

This case involved the crystal structure of a uranyl com­pound: such com­pounds contain O=U=O units that invariably are linear (*i.e.* in which the two O atoms are mutually *trans*). Surprisingly, one crystal structure of a polymeric uranyl com­pound (solved and refined in the non­centrosymmetric space group *Ama*2) was claimed to contain a *cis*-uranyl group, in which the O—U—O angle was 69.5 (6)° (CILFAF). The article notes that this angle was ‘considerably smaller than the range (95–105°) that is typically observed in transition-metal dioxido complexes’ (Vaughn *et al.*, 2007 ▸), which already is chemically implausible. The com­pound had been made from the reaction of a uranyl salt with ferrocenyl­carb­oxy­lic acid, and not only was the uranyl group *cis*, the carboxyl­ate groups had rearranged to generate one ferrocene unit and one ferrocenyldicarboxyl­ate (fcfc) group. The article also includes the highly implausible suggestion that this migration of carboxyl­ate groups between ferrocenyl units was dynamic on the NMR time scale in solution. Outside of carbonium ion chemistry (which is not relevant to this uranium com­pound), dynamic and reversible breaking of C—C bonds under such mild conditions is without precedent.

A group skeptical of the *cis*-uranyl geometry was unable to grow crystals of the original com­pound, but they showed that a closely related com­pound (BEFPEJ) had a *trans*-uranyl geometry, as expected (Villiers *et al.*, 2008 ▸). Without access to the original reflection intensities (which were not deposited in the CSD), we cannot say for certain what crystallographic error(s) were committed. There are several possibilities, such as the chosen unit cell was too small because some weak reflections were missed, or the chosen space group was wrong, or the atomic positions refined to a false minimum. This latter phenomenon is particularly likely when com­pounds that contain heavy atoms (of which uranium is the epitome!) are refined in noncentrosymmetric space groups (Murphy *et al.*, 1995 ▸, 1998 ▸; Kuchta & Parkin, 1998 ▸).

**Lesson:** whenever a structural model is proposed that contradicts a large body of earlier work, it is essential to search diligently for alternative, more conventional, models that agree not only with the crystallographic data but also with structural and chemical precedent. Such searches are particularly crucial in situations known to require care, such as the refinement of structures having heavy atoms in noncentrosymmetric space groups.

## Conclusions

We hope that these examples will help to allay the aura of infallibility that crystal structures sometimes assume in the chemical community. Even if crystal structure models are deduced automatically by software, the results are inter­preted by humans. Crystallography is not an exact science: there is no magical formula that infallibly enables us to deduce the correct structure from the experimental data. Instead, one must combine crystallographic skill with chemical knowledge drawn from the entire field of chemistry: reaction chemistry, bonding theory, literature precedent (or lack thereof), *etc*. This goal entails hard work, but there is no good excuse for laziness. One crystallographer put it this way: ‘Investigators should take adequate pains to ensure that [their] results are correct. Any other course is indefensible’ (Marsh, 1995 ▸).

A principal take-home lesson from this article is that *checkCIF* is a great program, but it is entirely possible for a completely incorrect model to have a flawless report, and for a completely correct structure to give a report that contains lots of alerts (for example, because it does not diffract strongly, or it is not possible to apply a good absorption correction). This situation reflects a fundamental limitation of current automated structure-checking packages: they are numerically competent but chemically unsophisticated. Their use must be augmented by an analysis of the model by a knowledgeable and skilled chemist.^4^

But even for the expert, it can sometimes be difficult even to recognize that there is a problem in the first place; after all, some of the mistakes above were made by very competent scientists. It is always necessary to evaluate crystallographic models with a high degree of skepticism, and to avoid being complacent. For example, we can imagine that some will read the current article, understand how the crystallographer/chemist went wrong in the examples given, and conclude that similar mistakes will not happen to them. Those who come to that conclusion, however, should ask themselves the following: if presented with the original incorrect model, would they have been able to diagnose the error without having read the analyses above.

Wrong structures are more likely if there are problems with the crystal, such as poor resolution, weak intensities, disorder, twinning, pseudosymmetry, *etc*. With good data and a skilled crystallographer/chemist, the chance of a major error is almost zero. With bad data and a skilled crystallographer/chemist, or good data and an unskilled crystallographer/chemist, there is a small chance of a major error. With bad data and an unskilled crystallographer/chemist, a major error becomes rather likely. Although we cannot exert much control over the quality of data, we hope this article helps to increase the skill of those who practice the art.

## Footnotes

1 A related type of noncrystallographic chemical error is failing to recognize that diastereomers have cocrystallized. The camphor derivative **5c** (LOY­MER) in Grošelj *et al.* (2002 ▸) is a good example. The article reports the crystal structure of what is said to be the pure *endo* diastereomer, but in fact the crystal consisted of a 1:1 mixture of both the *endo* and the *exo* diastereomers.

2 We can assume that the mis-assignment of the identities of many of the atoms was an honest mistake. What cannot be forgiven, however, is that the article reports a microanalysis with carbon and nitro­gen percentages that agreed perfectly with the original (*i.e.* incorrect) formula. One can only conclude that the microanalytical data were entirely fabricated.

3 The story continued through several more chapters and to avoid taxing the reader, we cite here the corrections that followed: (*a*) Pluth & Smith (1982 ▸); (*b*) Pluth & Smith (1983*a* ▸); (*c*) Pluth & Smith (1983*b* ▸). There are several other similar claims of highly unusual species in zeolite A materials that are likely errors but not explicitly rebutted: (*d*) Firor & Seff (1977*b* ▸); (*e*) Firor & Seff (1977*c* ▸); (*f*) Firor & Seff (1977*d* ▸); (*g*) Firor & Seff (1978 ▸); (*h*) McCusker & Seff (1978 ▸); (*i*) Kim & Seff (1978 ▸); (*j*) Kim *et al.* (1977 ▸); (*k*) Kim *et al.* (2011 ▸).

4 Some good advice is given in the section ‘Is it Good Science?’ in the article by Legrand (2021 ▸).
